# Risk‐Taking Patterns of Children, Associated Cognitive Weaknesses, and Prevention of Negative Outcomes

**DOI:** 10.1176/appi.prcp.2020.20190020

**Published:** 2020-09-09

**Authors:** Ahmet Esat Imal, Sean O’Leary, Bruce E. Wexler

**Affiliations:** ^1^ Department of Pediatrics New Haven Connecticut; ^2^ Department of Psychiatry New Haven Connecticut; ^3^ Yale University C8 Sciences New Haven Connecticut; ^4^ Connecticut Mental Health Center New Haven Connecticut

**Keywords:** Executive functioning, Balloon Analogue Risk Task, Risk taking, Focused attention, Response inhibition, working memory

## Abstract

**Objective:**

Accidents, drug use, and unsafe sex associated with greater propensity for risk‐taking are leading causes of illness and death among adolescents. This study aimed to help identify and further characterize children with maladaptive risk‐taking to improve primary prevention interventions.

**Methods:**

Two scores from the Bubblegum Analog Risk‐Taking Task for Children (BART‐C), total points and average inflations of unpopped bubbles, were used in a cluster analysis to identify distinct patterns of risk‐taking among 6,267 kindergarten through eighth‐grade children. Clusters were compared with the Flanker Test of Focused Attention, the Go/No‐Go test of inhibition, and the List Sorting Working Memory Test.

**Results:**

Both BART‐C scores made significant (p<0.001) contributions in defining three clusters of children: reckless, risk avoidant, and adaptive risk‐taking. Clusters differed significantly on Flanker Test measures of incongruent accuracy (p=0.004) and reaction time (p<0.001), Go/No‐Go inhibition (p=0.001), and List Sorting Working Memory Test scores (p<0.001). The reckless cluster had lower Flanker accuracy and Go/No‐Go inhibition than did the other groups and lower working memory than the adaptive risk‐taking group. Compared with adaptive risk‐takers, the risk‐avoidant group was slower (p<0.001), showed a nonsignificant trend toward greater accuracy on the Flanker test, and had lower working memory scores (p<0.001).

**Conclusions:**

The BART‐C defined two maladaptive risk‐taking clusters: reckless and risk avoidant. Significant differences in cognitive function between these groups and the adaptive risk‐taking group provides external validation of and further characterizes the clusters. Early intervention may prevent future health‐compromising behaviors among reckless children and may promote fuller learning and development among risk‐avoidant children.

## HIGHLIGHTS

- Risk‐taking is a part of adaptive human development but may evolve to maladaptive risk‐taking with detrimental effects on future health and well‐being.
- Distinct patterns of maladaptive risk‐taking (i.e., recklessness and risk‐avoidance) are significantly associated with cognitive weaknesses.
- Identifying children with different maladaptive risk‐taking behaviors and cognitive weaknesses can help primary prevention programs reduce risk‐associated behaviors with negative health consequences.

Intrinsic mechanisms of self‐regulation of action, emotion, and cognition begin to develop early (1, 2) and are important for academic, employment, and health outcomes throughout life. For example, self‐regulation explains unique variance in reading and math ability in kindergarten (3) and predicts academic success throughout elementary school (4, 5, 6). Moreover, lower self‐regulation during childhood is associated with greater drug and alcohol use, legal problems, lower income, and poor cardiovascular health at ages 26–32 (7). It is important, therefore, to identify factors associated with poor development of self‐regulation.

Researchers have defined top‐down and bottom‐up components of self‐regulation, which influence each other (8). The bottom‐up component, also called a reactive process, is a rapid response to stimuli without cognitive effort. These bottom‐up processes can be regulated by deliberate top‐down processes, which respond to stimuli more slowly and require cognitive functioning. One aspect of these top‐down processes is executive functioning, which includes working memory, focused attention, and response inhibition (9). Poor self‐regulation and maladaptive risk‐taking can result when top‐down and bottom‐up processes develop in improper balance (8).

Risk‐taking is a part of human nature, and evolutionary theorists view the learning process of risk‐taking as part of adaptive human development (10). However, when children become more sensitive to reward and sensations, risk‐taking can evolve into a maladaptive form through compromised self‐regulation. A recent publication by the Centers for Disease Control and Prevention (CDC) (11) showed that the leading cause of death among children and adolescents is injury, accounting for 70% of the overall childhood and adolescence mortality in the United States. Fatal injuries can be caused by reckless driving, drug use, and violence, all related to maladaptive risk‐taking (7, 12, 13). Other major causes of illness in adolescence—drug use and unsafe sex (14)—are also related to risk‐taking. This dramatic picture highlights the importance of understanding maladaptive risk‐taking and identifying children who are at risk.

Each year more than a billion dollars are spent on school‐based adolescent education programs designed to reduce risky behaviors, such as smoking, substance use, reckless driving, and unprotected sex (14). Unfortunately, evidence to support the effectiveness of these programs is limited (14). The latest release of the CDC’s National Youth Risk Behavior Survey (https://www.cdc.gov/healthyyouth/data/yrbs/results.htm) shows that although 85% of high school students received prevention instructions in school, 46.2% did not use a condom and 13.8% did not use any birth control method during their last sexual intercourse, and 19.5% of students used tobacco at least 1 day during the 30 days before the survey. These findings highlight the need for new approaches to prevent high‐risk behavior among adolescents, perhaps starting when children are younger.

The Balloon Analog Risk Task (BART) has been widely used to measure self‐regulation and maladaptive risk‐taking (15, 16, 17, 18, 19, 20, 21, 22, 23), and higher scores on this test have been linked with drug use among adolescents (15, 24) and smoking (16) among adults. However, scoring and interpretation of task performance on the BART have been debated. During the BART, participants incrementally inflate balloons by pushing the space bar or tapping the computer screen, stopping whenever they want to collect points based on how large they have made the balloon. However, the balloons pop at random, and the participant receives no points. Participants who push their luck and make the balloons larger before stopping to collect points are considered high risk‐takers.

The first complexity in interpretation arises because very few participants inflate balloons to the extent that mathematical modeling shows is optimal for achieving the most points. The subjective risk tolerance of nearly all participants limits their reward potential. In other words, participants considered high risk‐takers compared with their peers may be operating at a rational strategy to optimize reward. For this and other reasons, researchers have considered several ways to score the test. Some researchers have used the sum of points from all unpopped balloons (total score) to evaluate performance (19, 20, 22, 23, 24). This measure is problematic because one participant may get a total score of 100 from two unpopped trials inflated to the maximum of 50 points while popping all the other balloons, and another may receive the same total score by inflating 10 balloons to 10 points and then collecting no more points. These results represent two different behavioral risk‐taking patterns. The other widely used measure is the average inflation of unpopped trials (adjusted score) (15, 16, 17). This score would clearly differentiate between the two participants just described, but by itself it does not distinguish between children who arrive at the same average through different strategies resulting in different overall scores. For example, one child may consistently inflate balloons to 10 points, with 25 not popping (total score 250), and another child may achieve the same adjusted score of 10 points with five inflations to 15, five inflations to 5, and the rest popping (total score 100). In a previous report, Bell et al. (25) addressed these problems in part by using a maladaptive risk‐taking or “recklessness” score, defined as the average of unpopped trials (adjusted score) minus the total points earned (total score). Children who had higher adjusted scores and lower total scores than their age‐matched peers were characterized as using a maladaptive risk‐taking strategy (25).

In this article, we further evaluate approaches to the scoring and interpretation of BART responses by using a cluster analysis based on simultaneous consideration of age‐adjusted z‐scores for both total and adjusted scores, with the goal of identifying distinct risk‐taking styles that characterize different groups of children. Using a slightly modified version of the BART for children (BART‐C), we applied this approach to a sample of 6,267 children from kindergarten through eighth grade for whom we also had assessments of executive function. The sample included but was 50% larger than that of our previous report (25). Our goal was to determine if it was possible to identify and characterize children with maladaptive risk‐taking in elementary school to make possible targeted interventions that might help prevent maladaptive risk‐taking behaviors in adolescence. We aimed to answer the following questions: What kind of risk‐taking behavioral patterns can be identified using BART‐C‐defined clusters? Do children in BART‐C‐defined clusters differ in executive function, thus providing external validation of the clusters and helping to inform possible targeted intervention?

## METHODS

### Participants

We analyzed archival data of 6,267 students from kindergarten through eighth grade, across 344 U.S. schools in 47 states, who participated in the ACTIVATE cognitive training between 2016 and 2019. All schools had purchased the ACTIVATE program produced and distributed by C8 Sciences, a startup company affiliated with Yale University. Of the schools, 70% were in low‐income areas, defined as having more than 50% of students eligible for free or reduced lunch according to U.S. Department of Education data. At the beginning of the training, students completed the BART‐C and three cognitive assessments in their classrooms. Scores from students with valid assessments (validity criteria below) on all measures were included in the analyses. Because school officials selected the program for use in their curriculum on the basis of previous research and because all data analysis was on the group level without identifying information about individual children, the Yale University Human Investigations Committee, the institution’s review board, determined that individual consent for the participants was not needed.

### Cognitive Assessments

After two initial sessions to allow students and teachers to get used to the executive function training program, students underwent computerized attention, self‐control, working memory, and risk‐taking assessments in classroom settings. Testing in a classroom rather than in a quiet office setting increases the test’s ecological validity. However, because there was no direct observation during the testing, embedded validity criteria were applied as a part of the autoscoring for all assessments (see below); 14% of the data were eliminated after validity criteria were applied.

#### BART‐C

This test of self‐regulation of risk‐taking was based on the Balloon Analog Risk Task developed by Lejuez et al. (18) but with the interface changed for children. In this adaptation, when the child clicks on an image of a monkey, the monkey puffs to blow up a bubble; more puffs generate larger bubbles and more potential points. Each bubble can increase incrementally to 50 puffs but may explode at any point. The child can stop enlarging the bubble at any time and click “save” to collect points based on the number of puffs and the size of the bubble. However, if the bubble bursts before the child stops enlarging it, no points are gained. There are 30 trials. Mathematically, the optimal number of puffs per trial is 38, but few children will consistently go that high. The test consists of three blocks of trials, and every participant gets the same sequences. An explosion at three puffs always occurs within the first five trials, so all children experience a bubble popping early in the task. In this study, two scores were generated: the total score, which was the sum of all points earned from unpopped trials, and the adjusted score, which was the average number of bubblegum puffs on the unpopped trials. At least four unpopped trials were required to create a valid adjusted score of an average of unpopped trials. Trials without any puffs were considered invalid. If a test had more than five such trials, the test was considered invalid.

BART‐C differs from the BART only in that a bubblegum bubble is made larger instead of a balloon. Reliability and validity have been demonstrated for the BART (17). BART‐C has the same number of trials as the BART and the same point system. In the BART‐C, we standardized the random explosion in the BART to ensure each child experienced a pop after only three puffs within the first five trials, and the following 25 trials were divided into sets of 12 and 13, each made up of trials that were set to pop after the same number of inflations for each child, although presented in a randomly generated order for each administration of the test. Bell et al. (25) demonstrated external validity of the BART‐C by showing systematic age‐related change in scores and associations with independent measures of executive functions.

#### The Flanker Test of Focused Attention

In this test, children indicate by keyboard response the direction (right or left) the center arrow points to in a linear horizontal array of five arrows. On incongruent trials, the four flanking arrows point in the opposite direction of the central arrow. Following the procedure described in the National Institutes of Health (NIH) Toolbox (nihtoolbox.org), we had 29 congruent trials and 17 incongruent trials. Because response times longer than 4,500 ms on incongruent trials and 3,500 ms on congruent trials are so slow as to suggest classroom distraction or momentary disengagement from the test, we excluded those trials. Because response times faster than 150 ms suggest random responding, we also excluded those trials. We considered tests having more than four incongruent “too slow” trials, more than seven congruent “too slow” trials, more than four “too fast” trials, less than 75% correct on the congruent trials, or fewer than eight correct incongruent trials to be invalid because there were not enough valid trials to reliably evaluate performance and because they suggested repeated distraction or disengagement. Finally, we considered tests invalid if average reaction time on correct incongruent trials was slower than 3,400 ms or faster than 250 ms or if scores demonstrated values more than two standard deviations from the mean indicative of outlier performance, general inattention to the test, or random responding. We used the percentage of correct responses in incongruent trials and average reaction time of correct incongruent trials as primary performance measures. Practice effects with repeated administrations were small (partial η^2^=0.009), and Pearson r between test and retest within 10 days was 0.67 (26).

#### List Sorting Working Memory Test

The List Sorting Working Memory Test followed procedures described in the NIH Toolbox, presenting a series of animals or household objects. In this test, the child must select the objects just seen from among a grid of 12 objects, clicking them in order from smallest to largest rather than the order in which they were presented. The test starts with a list of two objects. If the child completes the list accurately, the list length is increased by one. If the child errs, the same length list is repeated. Two failed attempts at the same list length end the test. The score is the sum of correct list lengths. The test has two parts. In part 1, trials of animals and household objects alternate. In part 2, animals and household objects are presented in the same trial, and the child must reorder the animals first and then the household objects. If a child was unable to accurately respond with a list of two objects, the test was considered invalid. In our study, the practice effects with repeated administrations were small (partial η^2^=0.005), and the Pearson r between test and retest within 10 days was 0.49 (26).

#### Go/No‐Go test

The Go/No‐Go test of response inhibition instructs the child to press the space bar whenever a “go” stimulus is presented but not when a “no‐go” stimulus is presented. There are three blocks with 50 stimuli (40 go and 10 no‐go trials), randomly ordered in sets of 10 with eight go and two no‐go stimuli in each set. Different go stimuli are used in each block; in the first block, “P” is the go stimulus, and “R” is the no‐go stimulus. In the second block this is reversed. In the third block, pictures of furniture are the go trials and pictures of foods, such as cake and ice cream, are the no‐go stimuli. Stimuli are presented for 400 ms with a 1,400‐ms response window after stimulus offset. Errors are indicated by display of a large red X. We eliminated trials with response times greater than 2,000 ms because the response occurred after presentation of another stimulus. We also eliminated trials with response times less than 150 ms because the response was too fast to confidently be related to the stimulus. We considered tests with less than 85% correct response to go trials invalid, because the child failed to establish the consistent response bias required to measure response inhibition. (Simply not responding because of general inattention or poor accuracy would also artificially elevate the rate of no‐go trials correctly skipped.) We also considered tests with more than 10 “too slow” trials or more than 15 “too fast” trials invalid, because of concern that the children were attending inconsistently or responding randomly. In this study, practice effects with repeated administrations were very small (partial η^2^=0.001), and the Pearson r between test and retest within 10 days was 0.72 (26).

### Data Analysis

We determined grade‐adjusted z‐scores for each child and performed k‐means cluster analyses to identify different patterns by using the BART‐C total and adjusted scores as factors, with two‐, three‐, and four‐cluster solutions compared with respect to meaningfulness of the functional patterns and degree of additional variance captured by additional clusters. On the basis of these criteria, the three‐cluster solution was selected and is presented below in the Results section. We evaluated the stability of the clusters by randomly assigning all participants to one of two subsamples (27). We evaluated differences in cognitive measures among the three clusters defined by BART‐C by using one‐way ANOVA with Tukey’s honestly significant difference post hoc comparisons. We set the threshold for significance at the 0.05 level for two‐tailed analyses. We conducted the analyses by using R, version 3.4.2, and SPPS, version 24.

## RESULTS

### Kinds of Risk‐Taking Behavioral Patterns Identified by Using BART‐C Clusters

**FIGURE 1:**
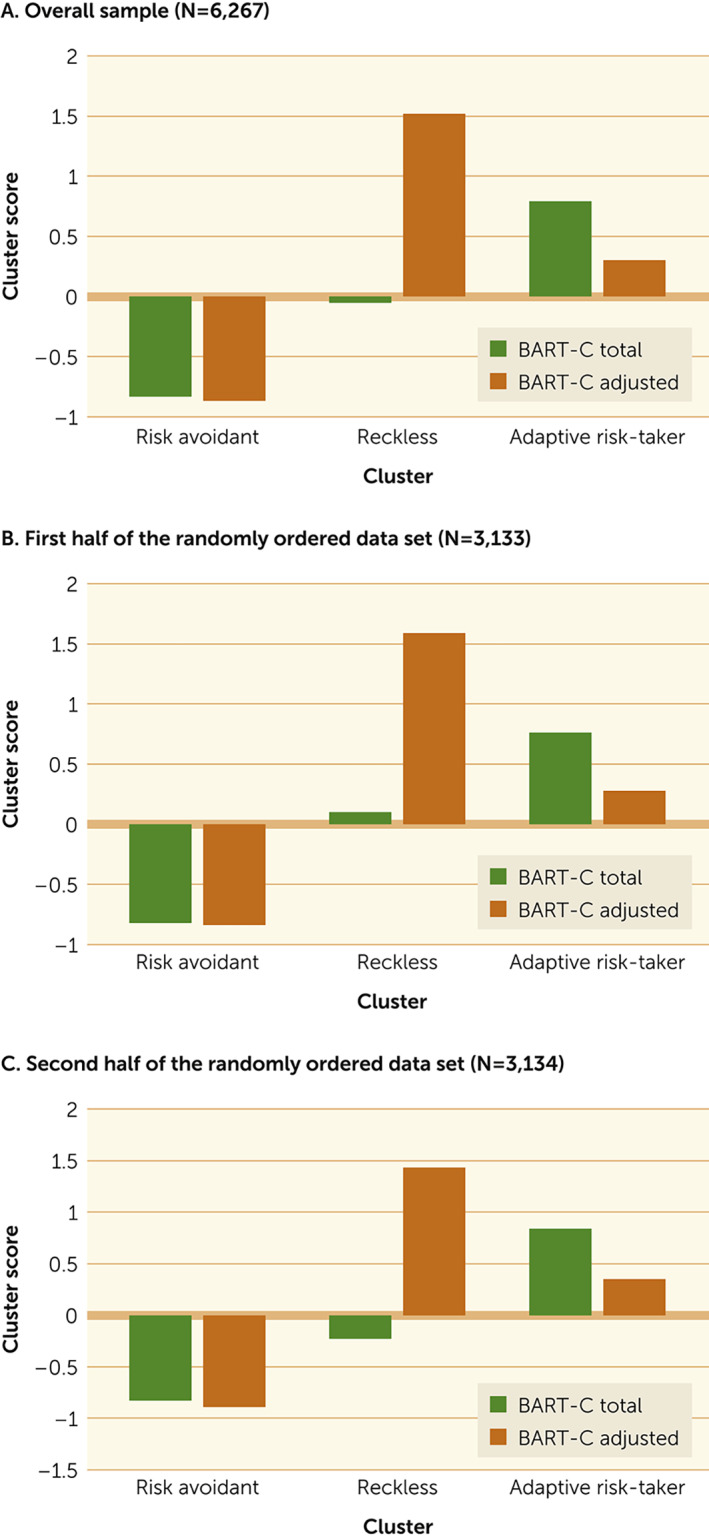
Contribution of total and adjusted Bubblegum Analog Risk‐Taking Task for Children (BART‐C) scores of 6,267 kindergarten–eighth grade children to cluster analysis, by risk‐taking behavior

Three clusters were defined using BART‐C total and adjusted scores (Figure 1). Both scores made significant contributions in determining cluster assignment (Table 1). Children in cluster 1 (N=2,585) were below average in both total and adjusted scores. These children (risk avoidant) very quickly stopped inflating the bubbles, had the most unpopped bubbles, but took or tolerated very little risk, and therefore had very low total scores. Children in cluster 2 (N=919) had by far the highest adjusted scores but the lowest number of unpopped bubbles and earned average total scores. These children (reckless) took great risks but with limited reward. Children in cluster 3 (N=2,763) achieved the highest total scores by taking moderate risks as reflected in intermediate adjusted scores (adaptive risk‐takers).

**TABLE 1:** Contribution of total and adjusted Bubblegum Analog Risk‐Taking Task for Children (BART‐C) scores to the cluster analysis of children showing risk‐avoidant, reckless, or adaptive risk‐taking behavior (N=6,267)

	Cluster		Error			
BART‐C score	Mean square	df	Mean square	df	F^a^	p
Total	1,505.92	2	0.44	5,315	3,459.35	<0.001
Adjusted	1,829.41	2	0.31	5,315	5,900.69	<0.001

^a^ df=2, 6,266.

### Differences in Cognitive Development Among BART‐C Clusters

**FIGURE 2:**
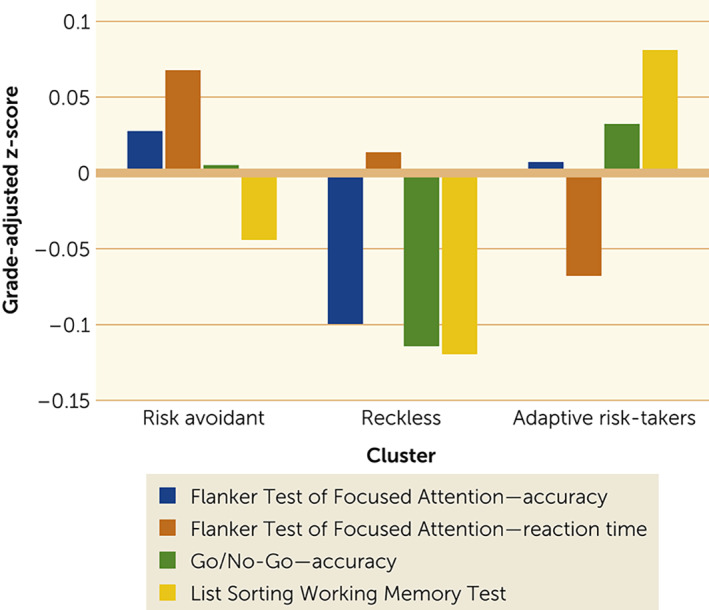
Executive functioning among 6,267 kindergarten–eighth grade children, by risk‐taking behavior

Analysis of variance showed that the effect of clusters was significant for all cognitive tests: Flanker Test accuracy (F=5.62, df=2, 6,264, p=0.004), Flanker Test reaction time (F=12.42, df=2, 6,264, p<0.001), Go/No‐Go test skip accuracy (F=7.50, df=2, 6,264, p=0.001) and List Sorting Working Memory Test (F=18.32, df=2, 6,264, p<0.001) (Figure 2). The children categorized as reckless had significantly lower incongruent trial accuracy on the Flanker test than did the children categorized as adaptive risk‐takers (p=0.014) or risk avoidant (p=0.003), lower self‐control on the Go/No‐Go task than did the children classified as adaptive risk‐takers (p<0.001) or risk avoidant (p=0.005), and lower List Sorting Working Memory Test score than the children classified as adaptive risk‐takers (p<0.001). The risk‐avoidant group was significantly slower than the adaptive risk‐taking group on the Flanker test (p<0.001) and showed a nonsignificant trend toward more accuracy and significantly lower working memory than did the adaptive risk‐taking group (p<0.001).

## Discussion

Decades of research have shown that maladaptive risk‐taking is linked to substance abuse (15, 24, 28, 29), injury (11, 12, 30), legal problems, and poor future health (7). Injuries, especially from motor vehicle accidents, are the leading cause of death among adolescents (12) and are associated with high risk‐taking. Consequently, some have suggested that risk‐taking by adolescents and young adults should be considered a public health issue (31). Identification of and appropriate intervention for children prone to maladaptive risk‐taking could constitute primary prevention of later poorly controlled high‐risk actions with significant personal and public health consequences.

The BART has been used to measure maladaptive risk‐taking, and higher scores have been associated with negative health consequences such as drug abuse and cigarette smoking (15, 16, 24). The task yields two different scores—total points earned and average number of inflations on unpopped trials—that, although correlated, can also represent different types of performance. In the present study, we used both scores together in a statistically unsupervised learning algorithm, cluster analysis, to identify possible distinct risk‐taking profiles in a large sample of school children. Both scores made significant contributions in identifying three distinct risk‐taking profiles. The cluster were highly stable in randomly generated subsamples.

Assessments of executive function in the same children provided external validation of the meaningfulness of the three clusters. The three BART‐defined subgroups differed significantly on the Flanker Test of Focused Attention, the Go/No‐Go test of response inhibition, and the List Sorting Working Memory Test. Children in the reckless and risk‐avoidant groups with the most extreme examples of the risk‐taking profiles defining each cluster also had the most pronounced cognitive characteristics associated with the cluster. Stability of the clusters in randomly generated subsamples provided further evidence of the reliability of the BART‐C scores in generating the clusters, and differences among clusters in all measures of executive function provided further external validation of the BART‐C scores as well as the clusters.

One subgroup was characterized by a very high average number of points on unpopped trials but very low total points earned; these participants blew up all the bubbles to high levels and most popped before the children stopped inflating them, earning them relatively few total points. The average inflations of the trials that did not pop, however, were very high. These children took a lot of risk for little reward. Their particular pattern was captured by subtracting their total score (which was low) from their adjusted or average inflations on unpopped trials (which was high). Bell et al. (25), in suggesting the value of this index, coined the term “reckless” risk‐takers, because these children took lots of risk for limited reward. The present study provided empirical confirmation of this group through a model‐free unstructured data analysis. Cognitive assessments confirmed their lack of self‐control; the reckless group had markedly lower inhibition of response on no‐go trials of the Go/No‐Go test. In addition, these children were less accurate on Flanker incongruent trials, consistent with less ability to inhibit response to the distracting incongruent arrows. They also had lower working memory, although this result may at least in part have been secondary to their inability to sustain attention during encoding. Children with these attributes may drive the relationship between high adjusted scores and real‐world risk behaviors reported previously (15, 17), but we did not measure real‐world risk behaviors in this study.

A second group of interest was characterized by a very low average number of points on unpopped trials and very few total points earned; these children were so risk avoidant that they consistently stopped inflating the bubble after only a few inflations. These children had the largest number of unpopped trials. The adaptive risk‐takers, in contrast, achieved the highest total scores and differed from the other groups by virtue of inflating the balloons to an intermediate level and having an intermediate number of unpopped balloons. Interestingly, the children in the risk‐avoidant group were also highly accurate but significantly slower in responding to the more difficult incongruent trials on the Flanker test than the children in the adaptive risk‐taker group, suggesting a general pattern of being very careful before responding. It is noteworthy that these children also scored significantly lower on working memory than the children in the adaptive risk‐taker group, but we do not have an explanation as to why this characteristic is part of their profile. It is possible that other psychosocial issues (e.g., anxiety) may be more prevalent among this group. It is also possible that lowered working memory in both the risk‐avoidant and reckless groups limited the children’s ability to learn during the BART‐C assessment and to develop more adaptive strategies. Both of these groups had lower total scores than did the adaptive risk‐taking group, consistent with an earlier report of an association between lower working memory scores and lower total scores (32).

Early intervention for the children in both the reckless and risk‐avoidant groups may be of significant long‐term value. As already described, reckless risk‐taking is associated with multiple negative health, social, and economic outcomes. On the other hand, high risk‐avoidance may limit one’s ability to take needed and modulated risks for learning (e.g., someone too afraid of falling will never learn to ski). By defining these two subgroups, it is possible to develop interventions that more specifically address different risk‐taking problems in the different subgroups of children and to use this knowledge of the cognitive characteristics of each group to inform the design of the interventions. These differences make apparent the limitations in both effectiveness and cost‐effectiveness inherent in many one‐size‐fits‐all interventions and provides direction for precision preventive medicine. Longitudinal studies will be needed to measure later real‐world risk behaviors in children with risk‐avoidant or reckless risk‐taking profiles in elementary school, as well as to determine the value of early and more targeted interventions in preventing maladaptive risk behaviors. We did not directly measure real‐world risk behaviors of the children in the present study, and instead have drawn from other studies which have shown connections between BART scores and real‐world risk behaviors.

## CONCLUSIONS

By using a novel approach to analysis of the BART, we identified three distinct patterns of risk‐taking behavior among elementary and middle school children. Two patterns, reckless risk‐taking and risk avoidance, were associated with distinct patterns of weakness in executive function. Both groups are probably at increased risk for a variety of negative outcomes. Identification of these groups early in school makes possible targeted interventions informed by children’s risk‐taking and cognitive profiles. Such primary prevention interventions are likely to be both more effective and more cost‐effective than the current one‐size‐fits‐all health and life skills programs used in schools today.
